# Psychodynamic university counseling: which factors predict psychological functioning after intervention?

**DOI:** 10.3389/fpsyg.2023.1134510

**Published:** 2023-05-10

**Authors:** Anna Maria Speranza, Costanza Franchini, Maria Quintigliano, Silvia Andreassi, Mara Morelli, Silvia Cerolini, Chiara Petrocchi, Alexandro Fortunato

**Affiliations:** ^1^Department of Dynamic and Clinical Psychology, and Health Studies, Sapienza University of Rome, Rome, Italy; ^2^Department of Psychology, Sapienza University of Rome, Rome, Italy; ^3^Department of Developmental and Social Psychology, Sapienza University of Rome, Rome, Italy

**Keywords:** university counseling, effectiveness, university students, personality, affective problems, young people, anxiety, depression

## Abstract

**Introduction:**

University counseling centers represent important resources for personal development, and students are increasingly turning to them for help. The present study aimed at, first, evaluating changing in psychological functioning before and after a university counseling intervention and, second, exploring which psychological variables predicted the intervention outcome.

**Methods:**

For this purpose, 122 students who attended university counseling services were administered measures to assess personality traits, and measures to assess state variables - intended as contextual, rather than stable, alterations in functioning - such as anxiety, hopelessness and depression. Several Linear Mixed Models were performed to measure the differences between OQ-45 scores before and after the intervention (one for each OQ dimension and OQ total score); then, two steps of multiple regression analyses were performed.

**Results:**

Significative reductions between pre-test and post-test OQ-45 scores were found, highlighting increased levels of well-being; personality traits seem not to be predictive of the intervention outcome, while state variables significantly contribute to the psychological wellbeing improvement after counseling intervention.

**Discussion:**

Our findings highlight the importance of paying attention to the role of affective difficulties in predicting the counseling effectiveness.

## 1. Introduction

Interest in the mental health of university students is increasing, given its relation to serious social and psychological concerns. Empirical research has highlighted high rates of psychological distress among university students, especially concerning anxiety, depression, interpersonal difficulties, and suicidal ideation (Mackenzie et al., 2011; Cerutti et al., 2020). A wide body of research shows that prevalence rates for these conditions have increased over the past 50 years (Hunt and Eisenberg, 2010; Eleftheriades et al., 2020). Specifically, a recent systematic review reported the prevalence of depression among university undergraduate students as 25%, and thus considerably higher than that of the general population (12.9%) (Lim et al., 2018; Sheldon et al., 2021). Despite this evidence, among college students, general feelings of depression and sadness have been more frequently registered than depressive or anxious symptomatology (Eleftheriades et al., 2020). A possible origin of university students’ distress may be difficulties associated with the specific developmental stage of emerging adulthood (Riva Crugnola, 2017). Several developmental challenges associated with this life stage, including separation from the family, the establishment of new social relationships, long-term goal setting, and financial management can, in fact, contribute to the onset of anxiety, depression, and general psychological distress (Ghilardi et al., 2018; Biasi et al., 2020). The demand for psychological support from college students is increasing worldwide (Center for Collegiate Mental Health, 2020). According to Eleftheriades et al. (2020), this growing search for help may be related to a new awareness of the importance of mental health and a reduction in stigma around mental health issues; however, it may also reflect an increasing severity of mental illness in this population (Hunt and Eisenberg, 2010).

University counseling centers attempt to respond to students’ increasing mental health needs representing important resources for personal development (Biasi et al., 2017). Several studies have highlighted the efficacy of university counseling services in reducing psychological distress and improving overall mental well-being among university students (Bani et al., 2020; Cerutti et al., 2020). Stallman et al. (2016), for example, reported substantial improvements in anxious and depressive symptomatology and sense of belonging in a sample of students who had received university counseling services. Evidence of the efficacy of a university counseling service was also provided by Murray et al. (2016), who reported that 63% of a sample of university students who had received counseling showed notable improvements in their mental well-being, according to the Clinical Outcome in Routine Evaluation-Outcome Measure (CORE-OM; Evans et al., 2002).

Despite this evidence, there remains a paucity of research on the variables that might predict the effectiveness of university counseling services. According to Mahon et al. (2015), effectiveness evaluation tools are critical to addressing this gap. Biasi et al. (2020) administered the Minnesota Multiphasic Personality Inventory-2 (MMPI-2; Butcher et al., 1989) to a sample of university students who had requested counseling, to detect whether its dimensions contributed to predicting outcomes. They found that students who presented higher levels of the Psychopathic Deviate (PD) dimension at pre-test showed a greater benefit from the counseling treatment at post-test, measured using the Outcome Questionnaire-45 (OQ-45; Lambert et al., 1996; Lo Coco et al., 2008; Biasi et al., 2020).

Several studies have been conducted about the role of personality traits as predictors of psychotherapy effectiveness, in line with Thalmayer (2018), who has argued that, in order to develop more targeted treatments, it may be relevant to investigate the predictive role of personality traits on treatment outcome. In a recent meta-analysis, Bucher et al. (2019) showed that five-factor model personality traits, detected prior to intervention, predict therapy outcome in different ways. Specifically, lower levels of neuroticism were found to be predictive of more positive outcomes, while higher levels predicted worse outcomes. These findings could be related to the fact that factors associated with neuroticism, such as distress, anxiety, and demoralization, could have an impact on individual coping skills, thus influencing therapy. High levels of extroversion, instead, were found to be predictive of greater symptom reduction after treatment (Bucher et al., 2019). Moreover, even in these studies, personality traits were found to be differently associated with treatment outcome depending on the moderators. Treatment duration, for example, appeared to moderate the association between personality traits and treatment outcome. In the light of these considerations, it appears interesting to investigate the role of personality traits within the specific framework of university counseling.

Furthermore, with reference to state variables, Hall et al. (2018) analyzed the predictive role played by anxiety and depression in relation to the premature termination of university counseling interventions. They found that mild levels of depressive symptoms and higher levels of social anxiety were associated with a greater likelihood of completing the intervention, while more symptoms of generalized anxiety were associated with a lower likelihood of completion (Hall et al., 2018). The authors highlighted the need for empirically supported intervention strategies within university counseling services, particularly to benefit students with elevated anxiety and depression (Swift and Greenberg, 2012; Hall et al., 2018).

Following this evidence, it seems reasonable to assume that an exploration of the potential role played by certain trait variables (e.g., personality traits) and state variables (e.g., anxiety, depression, hopelessness) in predicting the outcome of a counseling intervention may provide an interesting contribution to the field. To the best of our knowledge, an empirical study conducted at Roma Tre University (Biasi et al., 2020) has examined the predictive role of personality traits and symptomatological dimensions on university counseling outcomes.

Accordingly, the present study aimed at evaluating the efficacy of a university counseling intervention and detecting which psychological variables predicted the treatment outcome. For this purpose, a sample of university students was recruited from the Counseling Center of Sapienza University of Rome. Prior to the counseling intervention, a consultant of the Counseling Center conducted a brief exploratory interview with each student, to explore their request. If it was suitable for the counseling service, the students were asked to complete a battery of questionnaires prior to their first session (i.e., pre-test). The entire counseling intervention comprised four sessions. With the exception of the trait measure, which was only administered pre-test, students were administered all other questionnaires a second time, after the fourth session (i.e., post-test). If students’ requests were not deemed suitable for the Counseling Center (e.g., because more specialized help was required), they were referred to the appropriate service (in the next paragraph, inclusion and exclusion criteria are described).

## 2. Methods, materials, procedure and hypothesis

The counseling intervention provided at Sapienza University of Rome is psychodynamic in its theoretical orientation. It primarily focused on exploring and reflecting on developmental issues specific to “emerging adulthood” (Arnett, 2000), which are often why students seek help. The intervention also involved a well-defined time frame: four sessions took place over 1 month, and a follow-up session took place approximately 3 months later. This established boundaries in the counseling setting, encouraging the students’ separation-individuation (i.e., a common challenge of young adulthood) (Ferraro and Petrelli, 2000). The intervention was guided by continuous reflection on the specific developmental difficulties of emerging adulthood.

Then, the present study had two main research goals. The first was to assess improvements in students’ psychological overall wellbeing before and after the counseling intervention. The second goal was to investigate which variables, among some personality traits and some state variables referring to anxiety, depression and feelings of hopelessness, predicted the greatest change in perceived well-being after the counseling intervention compared with baseline.

*Hypothesis 1*: The overall well-being of students, as measured using the OQ-45 (Lambert et al., 1996), would show statistically significant improvement from pre-test to post-test in all the dimensions measured by the scale and in the total score.

*Hypothesis 2*: Specific personality traits, measured pre-test using the Personality Inventory for DSM-5—Brief Form (PID-5-BF) (Krueger et al., 2013), would contribute to predicting the intervention outcome, as measured using the OQ-45.

*Hypothesis 3:* Specific state variables, such as depression (as measured using the Beck Depression Inventory-II; Beck et al., 1996), anxiety (as measured using the Beck Anxiety Inventory; Beck and Steer, 1993), and hopelessness (as measured using the Beck Hopelessness Scale; Beck et al., 1974; Perczel Forintos et al., 2013), would additionally contribute to predicting the intervention outcome.

The study participants were students who attended the Counseling Service of Sapienza University of Rome between March and September 2022. A total of 229 students attended the preliminary screening interview. Of these, 194 were referred to the counseling intervention and the rest were referred to specialist services (e.g., psychiatric services, psychotherapy, etc.). The exclusion criteria were: (a) engagement in ongoing therapy and (b) the presence of severe psychiatric psychopathology (i.e., psychotic and bipolar disorders). The second point refers to the fact that given the specifics of the setting, it was deemed more appropriate to refer students with a prior psychiatric diagnosis to more appropriate services. Regarding the mortality of the sample, 3 students dropped out prior to the intervention; the remaining 191 completed the intervention, and 122 of these (63%) completed both sets of questionnaires. Our sample, therefore, consisted of 122 students. Prior to starting the intervention, students were asked to sign an informed consent form and provide basic anagraphic information on an online form. Assessment was done through a Google Form that was sent to students at pre- and post-test. 21 clinicians from our university’s graduate schools were conducting the intervention. All clinicians had undergone specific training on counseling. They were not informed of students’ scores on questionnaires before the first interview nor were they informed afterwards. Interventions were delivered both in-person and online. All student forms and questionnaires were coded anonymously, for the purposes of privacy protection. The research was conducted in accordance with the Ethical Principles for Medical Research Involving Human Subjects (Declaration of Helsinki) and approved by the local ethics committee.

### 2.1. Personality Inventory for DSM-5-Brief Form (PID-5-BF)

The PID-5-BF (Krueger et al., 2013) is a self-report questionnaire that assesses five personality trait domains: Negative Affectivity, Detachment, Antagonism, Disinhibition, and Psychoticism. It was developed by extracting 25 items from the full set of 220 in the original PID-5 (Krueger et al., 2012). Each trait domain consists of five items, and each item is rated on a 4-point scale (ranging from 0 to 3). Higher scores represent greater dysfunction. Fossati et al. (2017) highlighted the instrument’s good internal consistency and test–retest reliability in a sample of Italian adolescents. In the present study, Cronbach’s α values were as follows: 0.53 for Negative Affectivity, 0.43 for Detachment, 0.49 for Antagonism, 0.58 for Disinhibition, 0.63 for Psychoticism.

### 2.2. Outcome Questionnaire-45 (OQ-45)

The OQ-45 (Lambert et al., 1996) is a self-report questionnaire that assesses overall functioning. It consists of 45 items (rated on a 5-point Likert scale) across three subscales: Symptomatic Distress (SD), Interpersonal Relations (IR), and Functioning in Social Roles (SR). Besides the three subscale scores, a total score is derived from the sum of all items. Total scores range from 0 to 180, with higher scores corresponding to greater levels of distress. For this instrument, the cut-off that divides the nonclinical population from the clinical population is a score higher than 64 (Lo Coco et al., 2008).

The OQ-45 total score is considered one of the most trustworthy indices of treatment outcome (Vermeersch et al., 2000; Lambert et al., 2004). An Italian validation study showed excellent internal consistency relative to the total score (Cronbach’s *α* = 0.92) and SD scale (Cronbach’s *α* = 0.89) and good internal consistency relative to the IR and SR scales (Cronbach’s α between 0.62 and 0.77) in a non-clinical sample (Chiappelli et al., 2008). In the present study, Cronbach’s α values (pre and post-intervention) were as follows: between 0.88 and 0.94 (SD scale); between 0.71 and 0.75 (IR scale); between 0.58 and 0.70 (SR scale).

### 2.3. Beck Depression Inventory-II (BDI-II)

The BDI-II (Beck et al., 1996) is a self-report questionnaire that assesses the severity of depressive symptoms in adolescents and adults. Its 21 items are rated on a 4-point Likert scale, with higher scores corresponding to more severe depressive symptoms. Depression ratings are “minimal” (0–10), “mild” (11–16), “borderline clinical” (17–20), “moderate” (21–30), “severe” (31–40), or “extreme” (41–63). In the present study, a cut-off of 31 was used to determine severe depression. The Italian version of the instrument has been shown to have excellent internal consistency (Cronbach’s *α* = 0.80) and test–retest reliability (*r* = 0.76) (Sica and Ghisi, 2007). In the Italian validation, the bifactorial structure of the instrument, which includes a cognitive and a somatic factor, was confirmed. The first refers to aspects such as guilt, self-esteem, sense of worthlessness, sadness, pessimism. The second includes aspects such as fatigue, loss of energy, irritability. In the present study, Cronbach’s α values (pre and post-intervention) were as follows: between 0.79 and 0.89 for the somatic factor; between 0.85 and 0.89 for the cognitive factor.

### 2.4. Beck Anxiety Inventory (BAI)

The BAI (Beck and Steer, 1993) is a 21-item self-report questionnaire that assesses the severity of anxious symptoms using a 4-point Likert scale, with higher scores indicating greater symptom severity. Anxiety ratings are “minimal” (0–7), “mild” (16–25), “moderate” (16–25), or “severe” (26–63). In the present study, a cut-off of 26 was used to determine severe anxiety. The Italian validation of the questionnaire, conducted on non-clinical and clinical samples, showed good psychometric properties (Sica and Ghisi, 2007). In the present study, Cronbach’s α values was 0.91 at pre-intervention and 0.94 at post-intervention.

### 2.5. Beck Hopelessness Scale (BHS)

The BHS (Beck et al., 1974) is a self-report questionnaire that assesses the sense of hopelessness with regard to three dimensions: feelings about the future, loss of motivation, and future expectations (Beck and Steer, 1993). The measure includes 20 items, which are evaluated as true or false. The scale is often used as a suicidal risk assessment tool, with scores of 9 or above considered indicative of suicidal risk. A total score is generated from the sum of all item scores. Following Cerutti et al. (2023), the present study used the short version of the questionnaire, which is comprised of only one item for each component of hopelessness: cognitive (“Things just will not work out the way I want them to”), affective (“My future seems dark to me”), and motivational (“There’s no use in really trying to get something I want because I probably will not get it”). As highlighted by Perczel Forintos et al. (2013) this version may be more useful in clinical settings. Further, the short version of the BHS has been shown to have excellent psychometric properties and internal consistency (Cronbach’s *α* = 0.85) (Perczel Forintos et al., 2013). In the present study, Cronbach’s α values was 0.40 at pre-intervention and 0.52 at post-intervention.

### 2.6. Statistical analysis

All statistical analyses were performed using Jamovi 2.3 (2022). Preliminary descriptive analyses were conducted to test if the dependent variable (OQ-45 total score) was different between males and females or in relation to age.

First, linear mixed model was performed to measure the difference, for each student, between OQ-45 scores at pre-test and post-test, considering “students” and the “therapists” as cluster variables, and the time (Pre-counseling and Post-counseling) as factor variable.

Second, multiple sets of regression analyses were performed. Initially, scores for each PID-5-BF dimension and the PID-5-BF total score were regressed on the change of OQ-45 total scores between pre- and post-counseling. A second step was then applied, adding the following independent variables to the PID-5-BF traits: BDI-II Somatic-Affective, BDI-II Cognitive, BAI total score, and BHS Short Form total score. All these variables have been considered in their change between pre- and post-counseling. Changes in *R^2^* were used to evaluate the predictive power of each model. Variance inflation factor (VIF) indicators were used to test the multicollinearity between variables.

## 3. Results

Regarding the characteristics of the sample, 77% were female (*n* = 94) and 23% (*n* = 28) were male. The mean age of the participants was 23.4 years old (*SD* = 4.3). Students’ level of study was as follows: 43% (*n* = 53) were enrolled in a bachelor’s degree program, 28% (*n* = 34) in a master’s degree program, 4% (*n* = 5) in a PhD program, and 25% (*n* = 30) in a single cycle program (i.e., 5 or 6 years). With regard to the field of study, the sample was composed as follows: 25% (*n* = 30) of the sample studied literature and philosophy, 16% (*n* = 20) mathematical, physical and natural sciences, 11% (*n* = 13) medicine and dentistry, 10% (*n* = 12) medicine and psychology, 10% (*n* = 12) political science, sociology and communication, 9% (*n* = 11) engineering, 7% (*n* = 9) pharmacy and medicine, 7% (*n* = 9) economics, 5% (*n* = 6) law.

To determine the severity of students’ psychopathological symptoms prior to the intervention, cut-off scores for the BDI-II and BAI were proposed, as follows: BDI-II scores above 31 were considered indicative of severe depression; BAI scores above 26 were considered indicative of severe anxiety; OQ-45 scores above 64 are indicative of high distress associated with an increased number of symptoms, interpersonal difficulties, and worse quality of life. In this regard, at pre-intervention, 30% of the sample presented with severe depression, 26% presented with severe anxiety and 74% presented with elevated discomfort related to symptoms, relationships, and functioning in social role. We therefore measured the extent to which these rates changed after the intervention. Scores above the cut-off decreased remarkably in all three dimensions at post-test: among the 37 students who were above the BDI-II cut-off before the counseling intervention, the 75.7% (*N* = 28) of them were below the cut-off after it (and among the 83 students who were already below the cut-off, only 1 was above the cut-off after the counseling); among the 32 students who were above the BAI cut-off before the counseling, 62.5% (*N* = 20) were below after it (and among the 86 students who were already below the cut-off, only 2 of them were above the cut-off after the counseling); finally, among the 90 students who were above the OQ-45 cut-off before the counseling intervention, the 42% (*N* = 38) were below the cut-off after the counseling intervention (and among the 30 students who were already below the cut-off, only 2 of them were above the cut-off after the counseling). Thus, we found a clinically significant change since among the students who were above the cut-off and thus fitting into a clinical population before counseling (Lo Coco et al., 2008), very considerable percentages were below the cut-off after it.

Table 1 presents the main demographic characteristics of the sample and the frequency of scores above the cut-offs at pre and post-test of the overall group of participants.

**Table 1:** Descriptive characteristics.

Students (*N* = 122)	*M_Age_* 23.4 *SD_Age_* 4.3	Percentage
	*N*	
**Sex**		
Female	94	77%
Male	28	23%
**Degree program**		
Bachelor’s	53	43%
Master’s	34	28%
PhD	5	4%
Single-cycle	30	25%
**Field of study**		
Literature and philosophy	20	16%
Mathematical, physical and natural sciences	13	11%
Medicine and dentistry	12	10%
Medicine and psychology	12	10%
Political science, sociology and communication	12	10%
Engineering	11	9%
Pharmacy and medicine	9	7%
Economics	9	7%
Law	6	5%
**Above the cut-off at pre-test**		
BDI-II	37	30%
BAI	32	26%
OQ-45	90	74%
**Above the cut-off at post-test**		
BDI-II	10	8%
BAI	16	13%
OQ-45	55	45%

BDI-II cut-off: >31; BAI cut-off: >26; OQ-45 cut-off > 64.

The ANOVA conducted to test if the dependent variable (OQ-45) was different between male and female participants has shown that there are no differences related to sex (*F* = 0.399, *p* = 0.529). Furthermore, the correlation between OQ-45 and age is not significant (*r* = 0.040, *p* = 0.535). Age and sex have been then not considered as covariates in the following analysis.

The first aim of the study was to detect whether there was an overall improvement in students’ mental well-being between pre-test and post-test, as measured using the OQ-45. We conducted three sets of Linear Mixed Models, for each dimension of OQ-45 and for the total score. As shown by the linear mixed model conducted on OQ-45 total score, the first hypothesis was confirmed: the OQ-45 total score would decrease between pre- and post- counseling (*F* = 62.6, df_1_ = 1, df_2_ = 121, *p* < 0.001), indicating greater well-being, even considering students nested within therapists (see Table 2). As indicated by the R-squared, the model explained the 63% of the variance. The ICC values of the cluster variables, were, respectively, 0.000 for “THERAPISTS” and 0.586 for “STUDENTS,” indicating that the 58.6% of the variance of the change between pre- and post- counseling OQ-45 total score is explained by the fact to have considered the multi-level data.

**Table 2:** Linear mixed model on OQ-45: fixed effects parameter estimates.

				95% Confidence interval				
	Effect	*B*	SE	Lower upper		df	*t*	*p*
OQ-45 total score	Post_counseling – Pre_counseling	−0.621	0.0785	−0.775	−0.467	121	−7.91	**< 0.001**
OQ-45 symptomatic distress	Post_counseling – Pre_counseling	−0.671	0.0754	−0.819	−0.523	121	−8.89	**<0.001**
OQ-45 interpersonal relations	Post_counseling – Pre_counseling	−0.264	0.0718	−0.405	−0.123	121	−3.68	**<0.001**
OQ-45 functioning in social roles	Post_counseling – Pre_counseling	−0.521	0.0984	−0.713	−0.328	121	−5.29	**< 0.001**

Significant values are in bold.

Even considering the dimension Symptomatic Distress of OQ-45, the second hypothesis was confirmed: SD would decrease between pre- and post- counseling (*F* = 79.1, df_1_ = 1, df_2_ = 121, *p* < 0.001), indicating greater well-being (see Table 2). As indicated by the R-squared, the model explained the 65% of the variance. The ICC values of the cluster variables, were, respectively, 0.000 for “THERAPISTS” and 0.610 for “STUDENTS,” indicating that the 61.0% of the variance of the change between pre- and post- counseling OQ-45 SD is explained by the fact to have considered the multi-level data.

Regarding the dimension Interpersonal Relations of OQ-45, our hypothesis was confirmed: IR would decrease between pre- and post- counseling (*F* = 13.5, df_1_ = 1, df_2_ = 121, *p* < 0.001), indicating greater well-being (see Table 2). As indicated by the R-squared, the model explained the 69% of the variance. The ICC values of the cluster variables, were, respectively, 0.030 for “THERAPISTS” and 0.678 for “STUDENTS,” indicating that the 67.8% of the variance of the change between pre- and post- counseling OQ-45 IR is explained by the fact to have considered the multi-level data and that the 0.03% of the variance of the model is explained well having considered the therapist variable.

Finally, with respect to the dimension Functioning in Social Roles of OQ-45, our hypothesis was confirmed: SR would decrease between pre- and post- counseling (*F* = 28, df_1_ = 1, df_2_ = 121, *p* < 0.001), indicating greater well-being (see Table 2). As indicated by the R-squared, the model explained the 41% of the variance. The ICC values of the cluster variables, were, respectively, 0.00 for “THERAPISTS” and 0.369 for “STUDENTS,” indicating that the 36.9% of the variance of the change between pre- and post- counseling OQ-45 IR is explained by the fact to have considered the multi-level data.

The second aim was to investigate which personality dimension and which changing in the state dimensions, measured before and after the counseling intervention, predicted the changing in the overall wellbeing. A stepwise multiple regression was used for this objective, considering the difference between OQ-45 total scores before the counseling and OQ-45 total scores after the intervention as dependent variables. Specifically, personality traits (as measured by the PID-5-BF) and changing in state variables (i.e., anxiety, depression, hopelessness; respectively measured by the BAI, BDI-II, and BHS Short Form) were considered as predictors. The VID indicators of all variables were < 3, indicating a lack of multicollinearity (O’Brien, 2007) (Table 3).

**Table 3:** Multiple regression results predicting the change in OQ-45 total score after counseling intervention.

Predictor	Estimate	SE	*t*	*p*	Stand. Estimate
Intercept	2.413	2.047	1.179	0.240	
PID_NegativeAffectivity	−0.731	1.535	−0.476	0.635	−0.0216
PID_Detachment	−0.944	1.735	−0.544	0.587	−0.0267
PID_Antagonism	−2.072	1.794	−1.155	0.249	−0.0513
PID_Disinibhition	−0.688	1.575	−0.437	0.663	−0.0194
PID_Psychoticism	0.775	1.637	0.474	0.636	0.0262
ΔBDI_Somatic	1.234	0.191	6.460	**<0.001**	0.3519
ΔBDI_Cognitive	0.916	0.244	3.760	**< 0.001**	0.2158
ΔBAI	0.429	0.102	4.186	**< 0.001**	0.2067
ΔBHS	6.454	1.189	5.427	**< 0.001**	0.2371

PID, Personality Inventory for DSM-5-Brief Form; BDI, Beck Depression Inventory-II; BAI, Beck Anxiety Inventory; BHS, Beck Hopelessness Scale Short Form. Δ, change between pre- and post- counseling. Significant values are in bold.

The results show that personality traits do not predict the change of OQ-45 between pre- and post-counseling (R^2^adj. = −0.01, *F* = 0.59, *p* = 0.71), while all the changes in depression, anxious and hopelessness states between pre- and post-counseling significantly predict the change of OQ-45 and then the improvement in wellbeing after counseling (R^2^adj. = 0.69, *F* = 62.58, *p* < 0.001). The model change is also significant (ΔR^2^ = 0.69, *F* = 138, *p* < 0.001).

## 4. Discussion

The present study sought to generate evidence of the effectiveness of university counseling services and to expand knowledge of which variables predict a better outcome for students who engage with such services. First, in accordance with previous studies, the present sample had more female than male students (Vescovelli et al., 2017; Cerutti et al., 2020). This may be explained by a more positive attitude toward psychological support and a greater tendency to seek it among women, relative to men (Elhai et al., 2008; Nam et al., 2010). However, as also found by Cerutti et al. (2023), no sex- and age-related differences were found in our study in measuring the overall well-being of OQ-45 total scores.

Regarding the first aim of the study, significant results were reported. The comparison of students’ distress levels between pre-test and post-test showed a relevant improvement in well-being, as measured by the OQ-45, which is a widely used and reliable measure of treatment effectiveness. First, our results revealed a decrease in the Symptom Distress (SD) dimension after the counseling intervention. The present findings are in line with numerous studies showing the effectiveness of university counseling services in reducing anxiety and depressive symptoms (Østergård et al., 2017; Barnett et al., 2021; Cerutti et al., 2022, 2023). Biasi et al. (2017), for example found, in a sample of students who had undergone counseling, not only a significant decrease in internalizing disorders such as anxiety and depression, but also a substantial improvement in all three dimensions of the OQ-45.

Even in our study, the decrease in the OQ-45 Total Score suggests that the counseling intervention acted not only on reducing initial symptomatology, but also on improving interpersonal relationships and functioning in social roles. In addition to the significant reductions found in the OQ-45 SD, all dimensions of the OQ-45 were, in fact, significantly reduced at post-test, indicating a probable effect of counseling also on self-image change and reduction of interpersonal problems. With respect to self-image, diminished scores in the Social Role (SR) dimension, indicate a lower degree of perceived difficulty in the role played in society (e.g., student) and greater social adjustment. This result appears intriguing as it highlights the effectiveness of college counseling also in the area of self-satisfaction, of self-efficacy and sense of belonging, further confirming evidence already attested in the literature on university counseling (Stallman et al., 2016; Bani et al., 2020). Since the SR dimension refers precisely to self-perception within one’s role in society, in fact, we may assume that counseling also reinforced students’ own perceptions of self-efficacy in university setting. Although these considerations would require further investigation, we know that university counseling was also found to be effective in improving perceived self-efficacy and, consequently, academic success (Talsma et al., 2018; Bani et al., 2020).

Regarding the Interpersonal Relations (IR) dimension, the decrease in scores at post-intervention is indicative of lowered difficulties related to conflict, interpersonal problems and loneliness. Interestingly, although this dimension showed a statistically significant improvement after counseling, the pre-post difference was less evident than for the others. We hypothesize that this could be related to the fact that this area relates to larger-scale issues than the others (e. g., conflicts with family members), which may be more difficult to target in this specific form of intervention. Vermeersch et al. (2004) in analyzing the degree to which the OQ-45 is sensitive to clinically significant changes, found that in university counseling, it is more able to capture change in symptom distress than in the other two dimensions. Referring specifically to interpersonal relationships, the authors point out that change in this area may be more difficult to observe in the context of a limited time frame such as that of this type of intervention. It is reasonable to assume that changes in more obvious aspects such as psychopathological symptoms are more easily identified in this kind of setting. However, these considerations need further investigations to be fully supported.

Regarding the second aim of the study very interesting results were obtained from the regression analyses. However, our hypothesis that personality traits predicted outcomes was not confirmed. Biasi et al. (2020) examining the role that certain personality traits measured at the pre-test exerted on the efficacy of counseling intervention, found that the Pychopathic Deviate dimension of the MMPI-2 predicted better outcomes. In addition to the findings of Biasi et al. (2020), in our study, no personality traits among Negative Affectivity, Detachment, Antagonism, Disinhibition, and Psychoticism, measured through the PID-5 BF at the pre-test, were related to outcome. As argued by Thalmayer (2018), it seems logical to assume that personality is associated with intervention outcomes. However, an interesting review by Bucher et al. (2019) highlighted that treatment duration moderate the link between personality traits and treatment results. In particular, the effect of personality traits seems to be heightened by longer duration. This may contribute to explain why within the limited duration of counseling, our results found that personality traits were not related to outcome.

With regard to our third hypothesis, we found that pre-post differences in somatic BDI, BHS, BAI and cognitive BDI scores all predicted the outcome. Differences between pre- and post- counseling in somatic depression and hopelessness scores were particularly predictive, highlighting the domain on which university counseling probably exerts its effect to a greater extent. This finding aligns with Cerutti et al. (2020), who showed that students reporting greater depression and somatic complaints at pre-counseling showed a more pronounced decrease in these symptoms at post-counseling.

University counseling can be assumed to have exerted its effectiveness restoring students’ sense of confidence and openness toward the future; in fact, university counseling services seem to be effective at not only reducing anxiety and depressive symptoms, but also relieving students’ sense of hopelessness. Although to a lesser extent, differences in pre- and post-scores of anxiety and cognitive depression also predicted the outcome of counseling. In other words, a greater discrepancy between initial and final measurements revealed a better counseling outcome. Interestingly, the cognitive factor of depression had less predictive power than the other state variables. This factor includes facets such as guilt, self-esteem and sense of worthlessness. It is likely, therefore, to assume that college counseling exerted greater efficacy in alleviating the aspects of depression more related to the affective domain.

Therefore, our results provide further support for the idea that increased attention to students’ affective difficulties, including their sense of hopelessness, seems to be a priority for many university counseling interventions (Vescovelli et al., 2017).

### 4.1. Clinical implications

An important clinical implication of the present findings is that university counseling interventions should give significant attention to students’ affective problems, and particularly depression and feelings of hopelessness. These conditions often present with moderate severity among college students and may be appropriately treated by psychodynamic interventions (Vescovelli et al., 2017). The relatively high prevalence of depression and feelings of hopelessness among university students may be connected to the life stage of emerging adulthood, which demands the acquisition of a new autonomous identity, which many youths find disorientating (Petrelli, 1996). In this vein, a psychodynamic counseling intervention may help students to revive identity acquisitions that, when hindered, may generate a depressive state and a lack of motivation. Thus, in addition to decreasing symptoms, university counseling services should also aim at supporting students in their efforts to overcome developmental obstacles. These considerations, however, would require to be substantiated by future research developments that further investigate what factors are able to explain the effectiveness of psychodynamic counseling interventions.

## 5. Conclusion

The present study suffered from several limitations, which should be taken into account. The first pertains to the small sample size, which may affect the generalizability of the results. The second concerns the absence of a control group, which represents a strong limitation from methodological point of view. About this, looking at future directions it could be valuable to compare the treatment group to a control (waiting) group, as Biasi et al. (2017) have done, so finding an increase of learning indicators, at the end of experimental treatment with respect to the waiting group.

Thirdly, with regard to the sample, the fact that more severe psychiatric diagnoses were referred to other services suitable for their treatment, is a limitation for the generalizability of our results to the entire population of university students. Another limitation relates to the use of self-report instruments, which entail a risk of social desirability. However, in a context such as university counseling (to which students spontaneously refer), the effect of social desirability might be mitigated by the need to provide a truthful picture of oneself. In the future, it might be useful to assess, by recruiting larger numbers of participants, whether there are differences between those who conduct counseling in person and those who participate online.

Nevertheless, the present study has also some important strengths because it provided new and significant evidence for the effectiveness of university counseling, in two respects. First, it found a significant decrease in initial symptomatology and psychological distress after a university counseling intervention. Second, it provided useful data for understanding which variables might be most predictive of counseling outcome.

## Data availability statement

The raw data supporting the conclusions of this article will be made available by the authors, without undue reservation.

## Ethics statement

The studies involving human participants were reviewed and approved by the Ethics Committee of the Department of Dynamic and Clinical Psychology, Sapienza, University of Rome (No. 0000320, 16/04/2020). The patients/participants provided their written informed consent to participate in this study.

## Author contributions

AS and AF: conceptualization of the study, writing, review, and editing. SA and AS: supervision and project administration. CF and MQ: analysis of data, methodology, and writing original draft. MM, SC, and CP: investigation. All authors contributed to the article and approved the submitted version.

## Conflict of interest

The authors declare that the research was conducted in the absence of any commercial or financial relationships that could be construed as a potential conflict of interest.

## Publisher’s note

All claims expressed in this article are solely those of the authors and do not necessarily represent those of their affiliated organizations, or those of the publisher, the editors and the reviewers. Any product that may be evaluated in this article, or claim that may be made by its manufacturer, is not guaranteed or endorsed by the publisher.
